# Efficacy and Safety of Broncho Muco Cleaner Balloon Dilation Therapy in Chronic Bronchitis-Predominant COPD: A Prospective Interventional Study

**DOI:** 10.3390/jcm15145607

**Published:** 2026-07-17

**Authors:** Erdoğan Çetinkaya, Mustafa Çörtük, Demet Turan, Umut İlhan, Fulya Senem Karaahmetoğlu, Zeynep Betül Özcan, Barış Demirkol

**Affiliations:** 1 Department of Pulmonology, Yedikule Chest Diseases and Thoracic Surgery Training and Research Hospital, Istanbul 34020, Türkiye; mcortuk@yahoo.com (M.Ç.); drdemetturan@gmail.com (D.T.); dr.umutilhan@gmail.com (U.İ.); 2 Department of Physiotherapy and Rehabilitation, Hamidiye Institute of Health Sciences, University of Health Sciences, Istanbul 34668, Türkiye; fulyakaraahmet@gmail.com (F.S.K.); zbetulozcan@gmail.com (Z.B.Ö.); 3 Department of Pulmonology, Basaksehir Cam and Sakura City Hospital, Istanbul 34480, Türkiye; barisdemirkol34@gmail.com

**Keywords:** COPD, bronchoscopy, quality of life, dyspnea

## Abstract

**Background/Objectives**: COPD is a chronic, progressive, heterogeneous disease. While volume-reducing procedures are available for advanced emphysema-predominant COPD, options remain limited for chronic bronchitis-predominant COPD. Broncho Muco Cleaner Balloon (BMCB) dilation therapy is a method that aims to provide clinical and functional benefit by causing damage to the goblet cells in the bronchial system. This study evaluated the safety and effects of this method on exacerbations, dyspnea and exercise capacity in chronic bronchitis-predominant COPD. **Methods**: In this prospective interventional study, a total of 35 patients with stage II–IV chronic bronchitis-predominant COPD underwent BMCB therapy. The results of 25 patients who completed the 12-month-follow-up period were analyzed. Dyspnea was assessed using the modified Medical Research Council (mMRC) scale, pulmonary function with spirometry, exercise capacity with the 6 min walk test (6MWT), and quality of life with the St. George’s Respiratory Questionnaire (SGRQ). **Results:** At the 12-month follow-up, there were significant improvements in mMRC scores (*p* < 0.001), moderate/severe exacerbations (*p* = 0.022), and in the symptom, activity, impact, and total scores of the SGRQ (all *p* < 0.05). However, no significant changes were observed in FEV_1_ (*p* = 0.291) or 6MWT distance (*p* = 0.420). No procedure-related mortality occurred during the study period. In one patient, bradycardia was observed during the procedure, but it did not cause hemodynamic instability. **Conclusions**: Based on the results of this study, BMCB dilation therapy in patients with chronic bronchitis-predominant COPD appears to be safe. It reduces the perceived severity of dyspnea, decreases the number of moderate/severe exacerbations, and improves quality of life, without causing significant changes in pulmonary function or exercise capacity.

## 1. Introduction

Chronic obstructive pulmonary disease (COPD) is a progressive and debilitating respiratory disorder characterized by persistent airflow limitation and chronic respiratory symptoms. Among its clinical phenotypes, chronic bronchitis—defined as chronic cough and sputum production lasting for a minimum of three months in two successive years—represents a particularly burdensome presentation due to its association with increased exacerbation frequency, decline in lung function, and impaired quality of life [1,2]. Exacerbations of chronic bronchitis-predominant COPD not only lead to recurrent hospitalizations and heightened healthcare costs but also significantly contribute to morbidity and mortality. Thus, interventions that can effectively reduce mucus hypersecretion and prevent recurrent exacerbations are of high clinical relevance.

The pathophysiology of chronic bronchitis is closely linked to goblet cell hyperplasia and mucus gland hypertrophy in the bronchial mucosa, resulting in excessive mucus production and impaired mucociliary clearance [3]. Conventional pharmacological treatments, including bronchodilators, corticosteroids, and mucolytics, provide symptomatic benefit but have limited impact on the underlying structural abnormalities of the airway epithelium.

Broncho Muco Cleaner Balloon (BMCB) dilation therapy has recently been proposed as a novel bronchoscopic intervention for patients with chronic bronchitis-predominant COPD. Unlike conventional airway balloon dilation, which is primarily intended to restore airway patency in stenotic lesions, BMCB therapy is specifically designed to achieve controlled mechanical interaction with the bronchial mucosa. The catheter incorporates a braided balloon that exerts a circumferential frictional force on the epithelial surface during controlled inflation and withdrawal. This mechanical effect is intended to produce superficial epithelial desquamation, particularly in mucus-producing epithelium characterized by goblet cell hyperplasia, while avoiding deep airway injury. The balloon does not incorporate thermal energy or any pharmacological or chemical coating; therefore, the proposed therapeutic mechanism relies solely on controlled mechanical epithelial abrasion. The subsequent epithelial regeneration is hypothesized to restore a healthier epithelial surface, reduce mucus hypersecretion, and improve mucociliary clearance. Although the biological rationale of this approach is promising, the available clinical evidence remains limited. Published evidence consists predominantly of uncontrolled studies and case series with relatively short follow-up periods, precluding definitive conclusions regarding long-term efficacy, durability, and optimal patient selection [4,5].

Because the mechanism of BMCB differs fundamentally from conventional balloon dilation procedures, its therapeutic objective is not simply airway expansion but controlled superficial epithelial remodeling. In addition to facilitating mucus clearance, limited airway lumen expansion following balloon dilation may further improve airflow dynamics. Preliminary clinical studies have suggested potential improvements in respiratory symptoms, exercise tolerance, lung function, and selected physiological parameters following BMCB therapy. However, these findings have been derived mainly from highly selected patient populations without randomized controlled comparisons, and confirmation in larger prospective studies with longer follow-up is still required.

To date, evidence regarding BMCB therapy has been generated primarily from a limited number of studies, highlighting the need for further prospective investigations to better define its safety profile, clinical effectiveness, and long-term durability in patients with chronic bronchitis-predominant COPD [4,5].

Therefore, this study aimed to investigate the safety profile of BMCB dilation therapy and its effects on the frequency of exacerbations, perceived dyspnea, and exercise capacity in patients with chronic bronchitis-predominant COPD.

## 2. Materials and Methods

### 2.1. Study Design

This study was designed as a prospective interventional study and was conducted at the Interventional Pulmonology Clinic of Yedikule Chest Diseases and Thoracic Surgery Training and Research Hospital. Participant enrolment began in February 2022, with the first bronchoscopic intervention performed in March 2022, and the study was completed in January 2025 following the 12-month follow-up of the last participant.

### 2.2. Study Population and Sample Size

Male and female patients aged 40–75 years with chronic bronchitis-predominant COPD were included in the study. Sample size estimation was based on the study by Karakoca et al. [6], in which a mean increase in Forced Expiratory Volume in 1 s (FEV_1_) of 0.49 within one month was considered clinically significant when comparing Visit 1 and Visit 5 measurements. Using a median difference of 0.49 and an interquartile range of 0.97, the required sample size was calculated to range from 27 to 44 patients for a power level between 70% and 90%. According to the power analysis performed with a paired *t*-test for Visit 1–Visit 5 comparisons, 33 patients were required to achieve 80% power at a 95% confidence level. To account for potential dropouts due to repeated measurements or other reasons, 35 patients were initially enrolled in the study.

Eligible participants fulfilled the following inclusion criteria: diagnosis of chronic bronchitis-predominant COPD (GOLD stage II–IV); age between 40 and 75 years; smoking cessation for at least six months; mMRC dyspnea score ≥ 2; clinically stable disease for at least three weeks before the procedure; optimized pharmacological treatment according to the GOLD 2022 recommendations; completion of an 8-week pulmonary rehabilitation programme before the intervention; and the ability to provide written informed consent.

According to the GOLD 2022 spirometric grading system, COPD severity was classified based on post-bronchodilator FEV_1_% predicted as grade II (50–79%), grade III (30–49%), and grade IV (<30%).

Detailed inclusion and exclusion criteria are presented in Supplementary Table S1.

### 2.3. Intervention and Procedure

All procedures were performed under general anesthesia. Because all participants had chronic obstructive pulmonary disease (COPD), no premedication was administered. Standard hemodynamic monitoring was applied, and preoxygenation was performed using a face mask before induction of anesthesia. General anesthesia was induced with intravenous midazolam (0.5–2 mg), fentanyl (2 μg/kg), propofol (2 mg/kg), and rocuronium (0.6 mg/kg). Airway management was achieved using an endotracheal tube with an internal diameter appropriate for fiberoptic bronchoscopy. Appropriate sealing techniques were employed to minimize air leakage around the bronchoscope during mechanical ventilation. Anesthesia was maintained with total intravenous anesthesia (TIVA) using continuous infusions of remifentanil (0.1–1 μg/kg/min) and propofol (4–6 mg/kg/h). At the end of the procedure, following recovery of adequate spontaneous ventilation and consciousness, neuromuscular blockade was reversed with intravenous sugammadex (1–2 mg/kg), when clinically indicated, and the patient was subsequently extubated.

A flexible video bronchoscope (Olympus BF-1TQ190; Olympus Medical Systems, Tokyo, Japan) with an outer diameter of 4.9 mm and a 2.0 mm working channel was used throughout the procedure.

A 120 cm braided balloon catheter with an outer diameter of 1.7 mm (Clinodevice, Istanbul, Türkiye) was introduced through the bronchoscope working channel. Braided balloons with diameters of 10 mm, 15 mm, and 20 mm were selected according to the bronchial diameter. Balloon inflation was performed using an electronic inflation pump (Clinodevice, Istanbul, Türkiye) with an inflation pressure ranging from 1.0 to 2.5 bar. The inflation pressure was selected according to the diameter of the target bronchus or bronchial segment to achieve adequate balloon expansion while minimizing excessive mechanical stress on the airway wall.

The braided balloon does not incorporate thermal energy or any pharmacological or chemical coating. Therefore, the therapeutic effect of BMCB therapy relies solely on the controlled mechanical interaction between the braided balloon surface and the bronchial epithelium. This controlled mechanical desquamation is intended to promote epithelial regeneration, reduce mucus hypersecretion, and improve mucociliary clearance.

The intervention consisted of three bronchoscopic sessions performed at three-week intervals under fluoroscopic guidance. Each procedure lasted approximately 60 min. During the first session, the right lower lobe bronchi were treated; during the second session, the left lower lobe bronchi were treated; and during the final session, both upper lobes were treated. Balloon dilation was performed sequentially in the lobar, segmental, and subsegmental bronchi.

**Figure 1:**
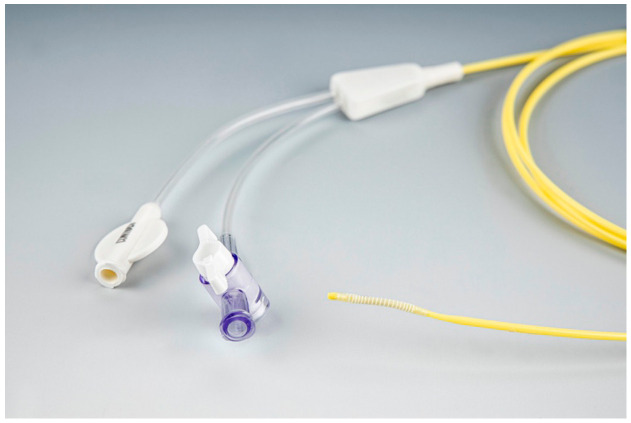
Braided balloon catheter.

**Figure 2:**
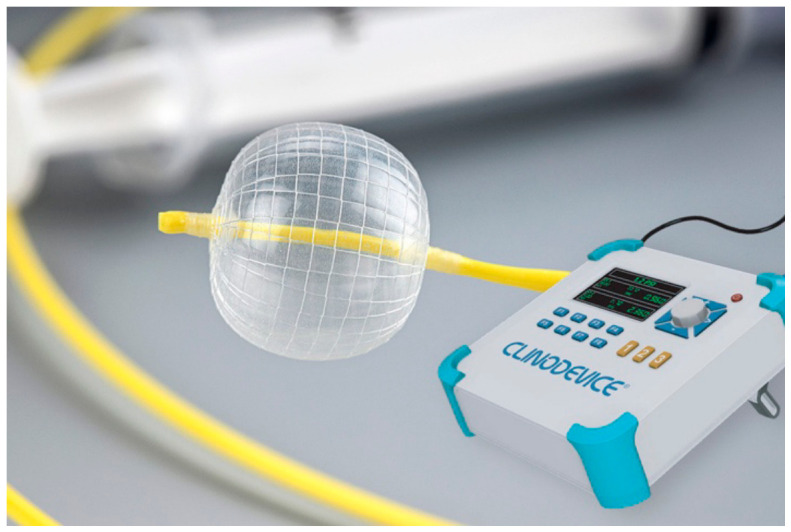
Electronic pump (1.0 to 2.5 bar) braided balloon catheter.

Representative images of the braided balloon catheter and the electronic inflation pump are shown in Figure 1 and Figure 2.

### 2.4. Study Assessments and Data Collection

According to the predefined study protocol, patients were evaluated at six scheduled visits (Visits 1–6), comprising three intervention visits followed by follow-up assessments at 3, 6, and 12 months after the final intervention.

The predefined primary outcome was the change in forced expiratory volume in 1 s (FEV_1_) from baseline to the 12-month follow-up. Secondary outcomes included changes in dyspnea severity assessed by the modified Medical Research Council (mMRC) scale, exercise capacity assessed by the 6-Minute Walk Test (6MWT), health-related quality of life assessed by the St. George’s Respiratory Questionnaire (SGRQ), the frequency and severity of exacerbations, and procedural safety.

Clinical assessments included the 6-Minute Walk Test (6MWT), pulmonary function testing, the modified Medical Research Council (mMRC) dyspnea scale, the St. George’s Respiratory Questionnaire (SGRQ), exacerbation assessment, and safety evaluation. However, because several participants missed one or more scheduled follow-up visits at 3 and/or 6 months, complete datasets at these intermediate time points were not available for all participants. Therefore, to ensure data completeness and consistency, the primary analyses presented in this manuscript were based on baseline (Visit 1) and 12-month follow-up (Visit 6) assessments of participants who completed the study.

The Six-Minute Walk Test (6MWT) was conducted in a 30 m corridor in accordance with the American Thoracic Society (ATS) recommendations. Patients were instructed to walk at their maximum possible speed, while oxygen saturation and heart rate were monitored. Dyspnea was assessed using the Modified Medical Research Council (mMRC) Dyspnea Scale [7]. Pulmonary function was evaluated with the MasterScope PC SFT device (Jaeger, Germany) in accordance with ATS guidelines [8].

The Saint George’s Respiratory Questionnaire, a disease-specific tool, was used to assess the effect of airway disease on general health status, daily activities, and perceived well-being. Scores range from 0 to 100, with 0 indicating no quality-of-life limitation and 100 reflecting the greatest degree of impairment [9].

Exacerbation is a critical concern for patients with COPD, carrying a risk of morbidity and mortality. It is anticipated that broncho muco-cleaner balloon treatment will reduce the frequency of exacerbations. To evaluate its effectiveness, the number and severity of exacerbations (mild, moderate, severe) were recorded at each follow-up visit. Exacerbation severity (mild, moderate, and severe) was defined according to the GOLD 2022 criteria.

The safety and reliability of the procedure were monitored for 12 months post-procedure, tracking any adverse events. During the study period, no procedure-related mortality occurred. However, one patient experienced bradycardia during the procedure, though it did not result in hemodynamic instability.

### 2.5. Statistical Analysis

In the analysis performed using SPSS 23.0 (IBM Corp., Armonk, NY, USA), statistical evaluations were made at 95% confidence levels. Differences between Visit 1 and Visit 6 were analyzed and appropriate statistical methods were applied according to the type of variables. Frequency and percentage distributions were reported for categorical variables, and the significance of differences between groups was evaluated using the Chi-square test. For continuous quantitative variables, descriptive statistics were presented by calculating mean, standard deviation, median, minimum and maximum values. Considering the number of observations and distributional characteristics of the data set, the analysis was performed using the nonparametric Wilcoxon test due to the skewness of the variables. This method was preferred in order to obtain more reliable results in data that do not meet the assumption of normal distribution. Because this was a single-arm pre–post interventional study in which each participant served as his or her own control, no multivariable adjustment for potential confounding variables (e.g., age, sex, smoking status, or comorbidities) was performed.

### 2.6. Ethical Considerations

This study was performed with the approval and supervision of the Istanbul University Clinical Research Ethics Committee (Decision Number: 2021-155) and conducted in accordance with the principles of the Declaration of Helsinki. It was registered at ClinicalTrials.gov (NCT05868941) and written informed consent was obtained from all participants.

### 2.7. Patient and Public Involvement

Patients and/or members of the public were not involved in the design, conduct, reporting, or dissemination plans of this study. Participants were recruited according to predefined eligibility criteria and received BMCB dilation therapy within the structured study protocol. The research question, study design, outcome measures, and analysis plan were determined solely by the investigators.

## 3. Results

A total of 35 patients with GOLD grade II–IV chronic bronchitis-predominant COPD were initially enrolled. During the follow-up period, four patients discontinued participation after the second intervention, four patients were lost to follow-up after completing the three intervention sessions, one patient withdrew consent, and one patient died during the sixth month of follow-up.

**Figure 3:**
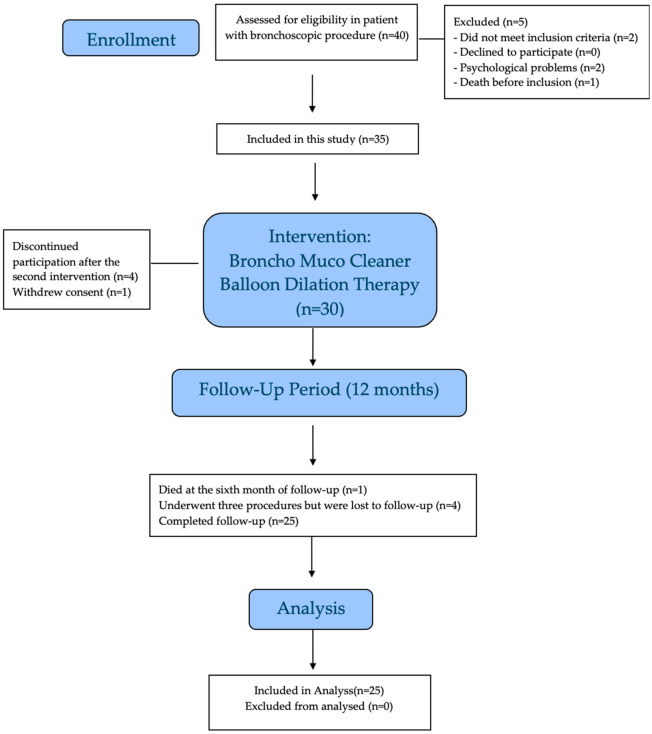
Study flow diagram.

Consequently, 25 patients completed the 12-month follow-up and had complete baseline and 12-month outcome data; therefore, they were included in the final analysis. The participant flow throughout the study is illustrated in Figure 3, and the demographic and baseline clinical characteristics of the analyzed cohort are summarized in Table 1.

The mean age of the analyzed cohort was 62.6 ± 7.8 years, and 80% were male. Most patients were former smokers (84%), while 16% had never smoked. In never-smokers, COPD was considered to be associated with non-tobacco risk factors, including long-term biomass exposure and/or occupational/environmental exposure, as documented in the clinical records. Hypertension was present in 36%, diabetes mellitus in 24%, arrhythmia in 12%, and sleep apnea in 8% of patients. Most patients were classified as GOLD grade III (72%), followed by grade II (20%) and grade IV (8%).

The pre- and post-treatment values of FEV_1_, mMRC, 6MWT, number of moderate/severe exacerbations, and SGRQ scores are presented in Table 2. Significant improvements were observed after treatment in dyspnea severity (mMRC, *p* < 0.001), number of moderate/severe exacerbations (*p* = 0.022), and SGRQ symptoms, activity, impact, and total scores (all *p* < 0.01). In contrast, no significant changes were detected in FEV_1_ (*p* = 0.291) or 6MWT distance (*p* = 0.420).

## 4. Discussion

Chronic bronchitis-predominant COPD is one of the most troublesome forms of the disease. Patients suffer not only from daily symptoms such as dyspnea, cough, and sputum production but also from frequent exacerbations, which reduce quality of life and increase the risk of hospitalization and mortality. Current pharmacological therapies alleviate symptoms to some extent but do not reverse the structural airway abnormalities, particularly goblet cell hyperplasia and mucus gland hypertrophy. This limitation has encouraged the development of bronchoscopic interventions targeting mucus hypersecretion. In the present prospective study, the predefined primary endpoint (change in FEV_1_) was not achieved. Nevertheless, BMCB dilation therapy demonstrated a favorable safety profile and was associated with clinically meaningful improvements in dyspnea, health-related quality of life, and exacerbation frequency, whereas pulmonary function and exercise capacity remained unchanged after 12 months. Therefore, these findings should be interpreted as suggesting potential benefits in clinically relevant secondary outcomes rather than evidence of improved airflow limitation. Given the non-randomized design, limited sample size, and relatively high attrition during follow-up, these observations should be interpreted cautiously and regarded as hypothesis-generating until confirmed in adequately powered randomized controlled trials.

Importantly, these findings should also be interpreted within the context of the current evidence base for BMCB therapy, which remains limited and is derived predominantly from uncontrolled studies and case series. Consequently, the present study contributes additional prospective clinical evidence but should not be regarded as providing definitive confirmation of treatment efficacy until validated by adequately powered randomized controlled trials [4,5].

Earlier studies on resector balloons, particularly those by Karakoca and colleagues, provided the first evidence that such interventions could be beneficial [10]. Their pilot study with ten severe COPD patients showed improvements in oxygenation, lung function, exercise capacity, and dyspnea scores without major complications. In a larger follow-up series of 188 patients, the same group reported significant gains in FEV_1_, walking distance, and quality of life, as well as a reduction in exacerbations [6]. These results positioned balloon dilation and curettage as a promising option for patients who remained symptomatic despite optimal medical therapy. However, these studies were uncontrolled and conducted in carefully selected patient populations, which limits the strength and generalizability of the available evidence [4,5].

Our findings partly overlap with, but also differ from, those reports. At one year, BMCB dilation therapy was associated with a significant reduction in dyspnea severity, fewer moderate-to-severe exacerbations, and a meaningful improvement in quality-of-life scores. These outcomes are important because they directly reflect the patient’s daily experience and have a strong link with prognosis. On the other hand, we did not observe significant changes in spirometric parameters or exercise capacity. This discrepancy may indicate that, while balloon dilation therapy helps by reducing mucus hypersecretion and improving airway clearance, it does not substantially alter fixed airflow limitation or the systemic factors that influence exercise performance.

This interpretation is biologically plausible because symptom burden in chronic bronchitis is influenced not only by fixed airflow obstruction but also by mucus plugging, impaired mucociliary clearance, and abnormal epithelial repair. Goblet cell hyperplasia contributes to excessive mucus production and airway luminal obstruction, whereas impaired mucus clearance promotes persistent inflammation and recurrent exacerbations. Consequently, interventions targeting airway mucus biology may improve dyspnea, health-related quality of life, and exacerbation frequency without necessarily producing parallel improvements in spirometric indices such as FEV_1_ [5,11].

Improved mucociliary clearance may also explain the reduction in exacerbation frequency observed in our cohort. More effective clearance of retained secretions may reduce mucus plugging, bacterial colonization, and chronic airway inflammation, thereby interrupting the mechanisms associated with recurrent exacerbations [11,12].

Similar clinical outcomes have also been reported with other bronchoscopic therapies targeting mucus hypersecretion, including bronchial rheoplasty and metered cryospray [13,14]. In the multicentre bronchial rheoplasty trial, goblet cell hyperplasia was reduced by 39%, accompanied by clinically meaningful improvements in CAT (−7.9 points) and SGRQ (−14.6 points) scores that were maintained through 12 months, although no significant changes were observed in pulmonary function. Likewise, metered cryospray therapy significantly improved SGRQ (−6.4 points), CAT (−3.8 points), and cough-related quality of life, whereas pulmonary function remained largely unchanged and the initial improvement in 6 min walk distance was not sustained at 12 months.

Despite employing different technologies, bronchial rheoplasty, metered cryospray, and BMCB therapy share a common therapeutic objective: selective modification of abnormal mucus-producing airway epithelium followed by epithelial regeneration. This shared biological target may explain the consistent improvement in patient-centred outcomes despite limited effects on pulmonary function observed across these bronchoscopic interventions [5,13,14].

Together with our findings, these studies suggest that bronchoscopic therapies targeting mucus hypersecretion primarily improve clinically meaningful symptoms and quality of life rather than reversing the fixed airflow limitation characteristic of COPD. Accordingly, the absence of a significant improvement in FEV_1_ in the present study should not be interpreted as inconsistent with the overall therapeutic concept of airway epithelial regeneration, although it remains an important negative finding that should be acknowledged when interpreting the results.

This observation is also consistent with previous reports indicating that symptom burden and health-related quality of life correlate only modestly with spirometric impairment in patients with chronic bronchitis-predominant COPD. Therefore, patient-centred outcomes may more accurately reflect the clinical benefits of mucus-targeted bronchoscopic interventions than spirometric measurements alone [12].

Although direct comparisons should be interpreted cautiously because of differences in patient populations, intervention techniques, and study designs, the consistency of symptomatic improvement across these bronchoscopic approaches supports the therapeutic potential of airway epithelial regeneration.

Another important aspect is safety. In our series, no procedure-related mortality occurred, and the only adverse event was a transient bradycardia that did not result in instability. These findings are consistent with earlier reports emphasizing the relative safety of this technique.

Another important consideration is the durability of treatment effects. Throughout the 12-month follow-up, we did not observe marked fluctuations in pulmonary function, dyspnea, exercise capacity, quality of life, or exacerbation frequency between the scheduled assessments. Nevertheless, a 12-month follow-up may not be sufficient to determine the long-term persistence of these benefits. This is supported by longer-term follow-up of other bronchoscopic interventions. For example, bronchoscopic thermal vapor ablation demonstrated the greatest improvement in lung function during the first 6–12 months, followed by a gradual decline during the second year, although values remained above baseline [15]. Although BTVA targets emphysematous lung tissue rather than mucus hypersecretion, these findings indicate that the durability of clinical benefit after bronchoscopic therapies should not be assumed. Therefore, studies with follow-up beyond 24 months are needed to determine whether the benefits of BMCB therapy remain sustained over time.

Overall, our findings suggest that the principal benefit of BMCB dilation therapy lies in improving clinically meaningful patient-centred outcomes—including dyspnea, exacerbation frequency, and health-related quality of life—rather than pulmonary function or exercise capacity. Given the substantial burden associated with chronic bronchitis-predominant COPD, these findings suggest potentially meaningful clinical benefits that require confirmation in adequately powered randomized controlled trials. Larger multicentre randomized controlled trials with longer follow-up are needed to confirm the durability of these benefits and to better identify the patients most likely to benefit from this intervention.

This study has several limitations. First, its single-centre, non-randomized design and relatively small sample size limit the generalizability of the findings. Second, the absence of a control group precludes definitive attribution of the observed clinical improvements solely to BMCB therapy. As pulmonary rehabilitation was part of the standard clinical management before the intervention, the potential residual effects of the 8-week pulmonary rehabilitation programme on the study outcomes cannot be completely excluded. In addition, participant attrition during the 12-month follow-up may have introduced selection bias. Therefore, the statistically significant improvements observed in the secondary outcomes should be interpreted with appropriate caution, as the relatively high attrition rate may have reduced the robustness of these analyses. Although a 12-month follow-up is appropriate for assessing procedural safety and short-term clinical outcomes, it may not be sufficient to evaluate the long-term durability of treatment effects. Previous bronchoscopic studies have shown that early clinical improvements may gradually attenuate beyond the first year. Therefore, longer-term follow-up studies are required to confirm the sustained efficacy of BMCB therapy. Furthermore, subgroup analyses according to blood eosinophil count, inhaled corticosteroid use, mucolytic therapy, and long-term oxygen therapy could not be performed because of the limited sample size and the incomplete availability of these variables for all participants. Finally, although the study was funded by Clinodevice, which also covered the article processing charge (APC) and provided congress participation support to two authors following acceptance of the study abstract, the sponsor had no role in the study design, patient recruitment, data collection, statistical analysis, interpretation of the results, manuscript preparation, or the decision to submit the manuscript for publication. Therefore, larger multicentre randomized controlled trials with appropriate control groups are warranted to confirm the efficacy and long-term safety of BMCB therapy.

## 5. Conclusions

The findings of this study suggest that broncho muco cleaner balloon dilation treatment may be a safe and promising therapeutic option for patients with chronic bronchitis-predominant COPD. The intervention was associated with improvements in perceived dyspnea, a reduction in moderate/severe exacerbations, and improved health-related quality of life, while no significant changes were observed in pulmonary function or exercise capacity. Larger multicentre randomized controlled trials are warranted to confirm the efficacy, safety, and long-term clinical benefits of this intervention.

## Figures and Tables

**Table 1:** Baseline demographic and clinical characteristics of the patients.

Variable	Mean
Age (year)	62.60 ± 7.75
BMI (kg/m^2^)	26.88 ± 6.40
Variable	*n* (%)
Sex	
Female	5 (20)
Male	20 (80)
Smoking Status	
Yes	0 (0)
No	4 (16)
Quit	21 (84)
Chronic Diseases	
HT	9 (36)
DM	6 (24)
Arrhythmia	3 (12)
Sleep Apnea	2 (8)
COPD Stage	
Stage II	5 (20)
Stage III	18 (72)
Stage IV	2 (8)

BMI: Body Mass Index, DM: Diabetes Mellitus, HT: Hypertension.

**Table 2:** Comparison of exacerbations, exercise capacity, pulmonary functions, dyspnea, and quality of life at pre- and post-treatment.

Parameter	Pre-Treatment (*n* = 25)	Post-Treatment (12 Months, *n* = 25)	*p*-Value
FEV1 (L)	0.99 ± 0.60	0.89 ± 0.43	0.291
mMRC			<0.001
Grade 0	0	1	
Grade 1	0	10	
Grade 2	5	11	
Grade 3	17	3	
Grade 4	3	0	
6MWT (m)	372.26 ± 101.43	373.44 ± 120.48	0.420
The number of moderate/severe exacerbations	2.24 ± 3.11	0.40 ± 0.95	0.022
SGRQ (point)			
Symptom score	68.47 ± 18.23	46.52 ± 20.28	<0.001
Activity score	81.47 ± 17.96	64.72 ± 19.74	0.006
Impact score	51.50 ± 13.73	33.93 ± 14.87	<0.001
Total score	63.53 ± 12.93	45.60 ± 15.33	<0.001

FEV1: Forced Expiratory Volume in 1 s, mMRC: modified Medical Research Council Dyspnea Scale, 6MWT: 6-Minute Walk Test, SGRQ: St. George’s Respiratory Questionnaire.

## Data Availability

The data presented in this study are available on reasonable request from the corresponding author. The data are not publicly available due to privacy and ethical restrictions.

## Supplementary Materials

The following supporting information can be downloaded at: https://www.mdpi.com/article/10.3390/jcm15145607/s1, Table S1. Eligibility criteria for study participation.

## Abbreviations

The following abbreviations are used in this manuscript:

ATS	American Thoracic Society
BMI	Body Mass Index
BMCB	Broncho Muco Cleaner Balloon
COPD	Chronic obstructive pulmonary disease
DM	Diabetes Mellitus
FEV1	Forced Expiratory Volume in 1 s
HT	Hypertension
mMRC	Modified Medical Research Council
PaCO_2_	Arterial partial pressure of carbon dioxide
SGRQ	St. George’s Respiratory Questionnaire
6MWT	6 min walk test
