# Supplementary material for: Efficacy and Safety of Broncho Muco Cleaner Balloon Dilation Therapy in Chronic Bronchitis-Predominant COPD: A Prospective Interventional Study
Source: J Clin Med. 2026 Jul 17;15(14):5607. doi: 10.3390/jcm15145607 (PMC13412491; doi:10.3390/jcm15145607)
Supplement: Supplementary file 1 [file jcm-15-05607-s001.zip › jcm-4322623-supplementary.pdf]

Supplementary Table S1. Eligibility criteria for study participation.

Inclusion Criteria	Exclusion Criteria
Adults aged 40–75 years, regardless of sex	Age <40 or >75 years
Diagnosis of chronic bronchitis-predominant COPD (GOLD stage II–IV)	Pregnancy or breastfeeding
Former smokers who had quit smoking for ≥6 months	Current smoking
mMRC dyspnea score ≥2	Concomitant asthma or positive bronchodilator reversibility
Receiving optimal pharmacological treatment according to the GOLD 2022 recommendations	Emphysema-predominant COPD
Clinically stable for ≥3 weeks before the intervention	Other clinically significant pulmonary diseases besides COPD
Completion of an 8-week pulmonary rehabilitation programme before the first BMCB procedure	Active pulmonary infection
Ability to understand the study procedures and provide written informed consent	Active malignancy
	Chronic kidney disease (estimated GFR <30 mL/min/1.73 m^2)
	Liver cirrhosis
	Clinically significant arrhythmia, left ventricular ejection fraction <45%, or pulmonary hypertension (estimated pulmonary artery systolic pressure >45 mmHg)
	Arterial blood gas abnormalities ($PaCO_2$ >55 mmHg or PaO_2 <55 mmHg on room air)
	Six-minute walk distance <100 m
	FEV_1 <15% predicted
	Continuous use of anticoagulants, clopidogrel, or equivalent antiplatelet therapy that could not be safely discontinued, or the presence of a bleeding diathesis
	Failure to receive regular LAMA/LABA/ICS triple inhaled therapy, when indicated according to GOLD recommendations
	Daily oral prednisolone dose >10 mg
	Previous lung volume reduction or other bronchoscopic lung device procedures (including endobronchial valves, coils, thermal vapor ablation, targeted lung denervation, or emphysema stents)
	Pneumothorax or pulmonary surgery within the previous 6 months
	Known hypersensitivity to medications required for bronchoscopy (e.g., lidocaine or benzodiazepines)
